# Is Use of Bone Cement for Treatment of Second Metatarsal Stress Fractures Safe? A Case Report

**DOI:** 10.7759/cureus.3436

**Published:** 2018-10-09

**Authors:** Haley M McKissack, Jun Kit He, Tyler P Montgomery, John T Wilson, Aaradhana J Jha, Leonardo V Moraes, Ashish Shah

**Affiliations:** 1 Orthopaedics, University of Alabama at Birmingham, Birmingham, USA; 2 Orthopaedic Surgery, University of Alabama at Birmingham, Birmingham, USA; 3 Orthopaedics, Massachusetts General Hospital, Harvard Medical School, Boston, USA; 4 Orthopedics, Instituto De Assistência Médica Ao Servidor Público Estadual (IAMPSE), São Paulo, BRA

**Keywords:** metatarsal, stress fracture, bone cement, second metatarsal, foot & ankle, metatarsal fracture, metatarsal stress fracture, second metatarsal stress fracture, second metatarsal fracture

## Abstract

Metatarsal stress fractures are common injuries of the foot and can be a source of chronic pain without appropriate management. Conservative management is first line, but surgery may be indicated in athletes, cases of nonunion, and fractures of the fifth metatarsal. We report a case of a 34-year-old female who presented to clinic for intractable pain of the left foot secondary to a stress fracture of the left second metatarsal, which had been previously treated with injectable acrylic bone cement. Calcium sulfate hydroxyapatite cement has a multitude of applications in orthopedic surgery, but to our knowledge no studies have documented its use in the treatment of metatarsal stress fractures. Our findings suggest that injectable calcium sulfate hydroxyapatite cement is not a suitable stand-alone treatment in fractures of the second metatarsal.

## Introduction

Stress fractures are common, spontaneous injuries resulting from a sum of individually harmless impacts. Their occurrence peaks between the second to fifth decades of life [1] and the majority occur in the lower extremities, with 8.8–25% reported in the metatarsals. Fractures of the second and third metatarsal are particularly common, contributing to 80–90% of sports-related metatarsal stress fractures [2]. The second and third metatarsals are less mobile than their medial and lateral counterparts and receive the majority of ambulatory stress [2]. These metatarsals have anchoring ligaments between their heads, which protect from fracture displacement but also increase the plantar-directed weight-bearing forces [3].

Stress fractures are thought to be caused by accelerated remodeling and microcracking from repetitive injury. Individuals participating in physical activities involving prolonged weight-bearing, recurrent motions, or generalized overuse are thought to be at increased risk [2]. Inadequate recovery time after such activities is also a concern. Additionally, individuals with conditions deleterious to bone strength and integrity are at increased risk [3].

Typically, all non-displaced metatarsal fractures and fractures of the second to fourth metatarsals displaced in the frontal plane without shortening of the respective ray can be treated non-operatively [4]. This begins with rest, ice, compression, and elevation with the goal to prevent progression to displacement or nonunion. Immobilization is not usually required and return to activity is typically at six to 12 weeks [2, 5]. If further stabilization is necessary, adhesive strapping and compression dressing with a wooden sole or a stiff-soled boot may be used [4]. For those at high risk of nonunion, internal fixation is indicated, with or without bone grafting [2].

## Case presentation

**Figure 1:**
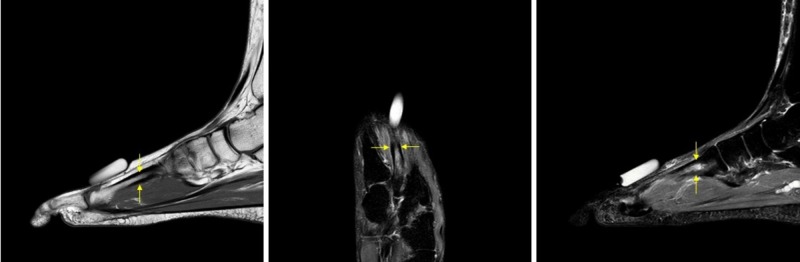
Magnetic resonance imaging (MRI) showing stress reaction of the second metatarsal prior to cement injection. From left to right: sagittal T1; Coronal STIR; sagittal STIR.

A 34-year-old female with no significant past medical history presented to our clinic after experiencing a left second metatarsal stress fracture (Figure 1). One year prior, while running errands around town, she suddenly felt a sharp pain in her left midfoot and promptly consulted an orthopedic surgeon who placed her in a boot. Six months later, after experiencing minimal improvement in her pain, a different orthopedic surgeon performed an open reduction and internal fixation by injecting 1 mL of bone cement into the diaphysis of the second metatarsal.

Over the next six months, she noticed no meaningful improvement in her pain. At this point, she presented to our clinic for a third opinion. During our initial visit with her, she stated that her left foot felt different than her right at baseline.

On physical exam, there was no gross deformity of her left lower extremity. The skin was intact with a healed incision over the dorsal midfoot, and there was point tenderness to palpation over the second metatarsal. Active and passive range of motion of the ankle and transverse tarsal joint was full and painless. Strength was 5/5 in dorsiflexion, plantarflexion, inversion, and eversion. Sensation to light touch was intact, Achilles reflex was present, and dorsalis pedis and posterior tibialis pulses were palpable.

**Figure 2:**
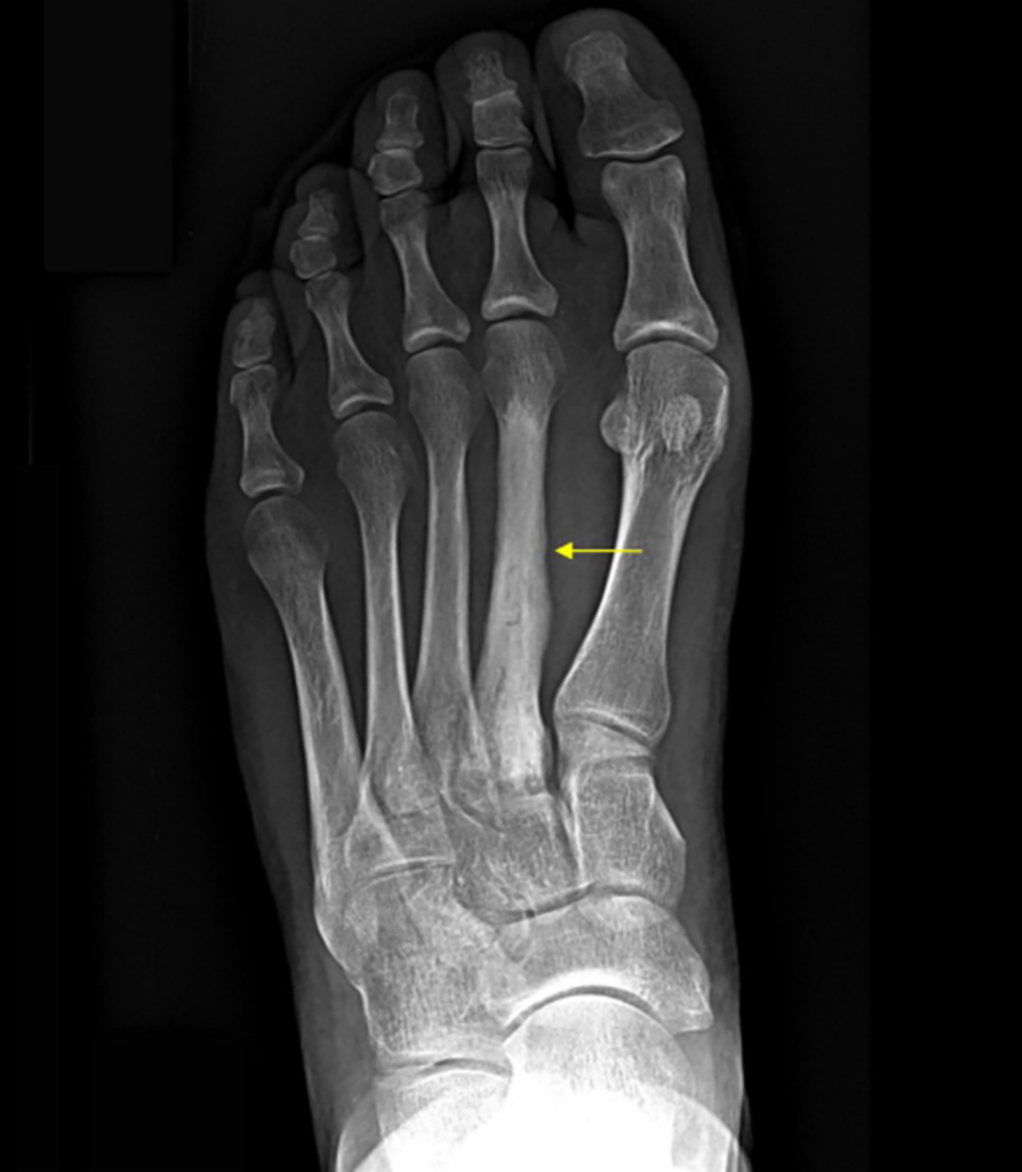
Pre-operative X-ray showing cement in the second metatarsal.

**Figure 3:**
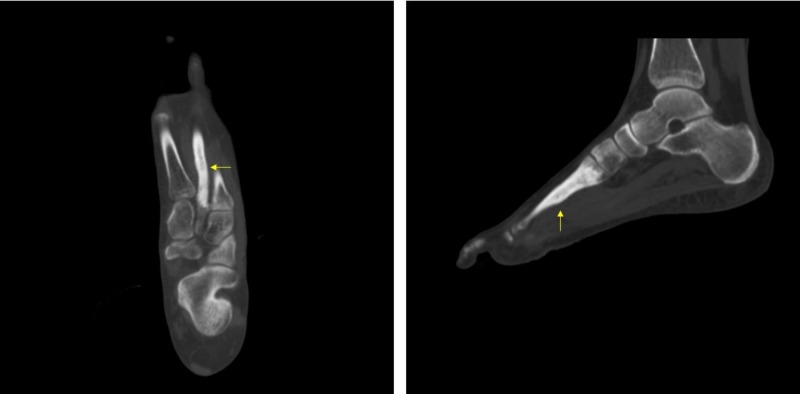
Pre-operative computed tomography (CT) showing cement in the second metatarsal.

**Figure 4:**
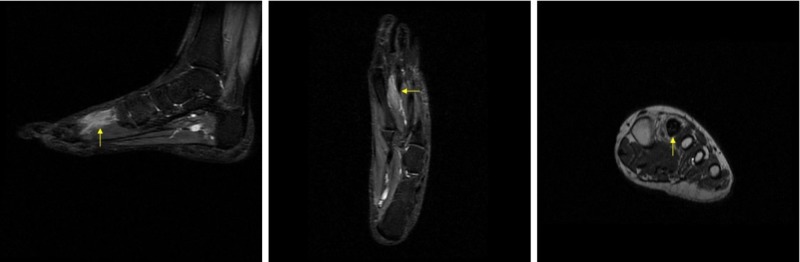
Pre-operative magnetic resonance imaging (MRI) showing cement in second metatarsal. From left to right: sagittal STIR MRI; coronal STIR MRI; axial T2 MRI.

Laboratory work revealed an elevated erythrocyte sedimentation rate of 36 (reference range: 0–20) and C-reactive protein of 34.74 (reference range: 0–10.9). Plain radiographs and a computed tomography (CT) scan of the left foot showed diffuse sclerotic changes and cement within the left second metatarsal (Figures 2, 3). Magnetic resonance imaging (MRI) showed diffuse edema of the left second metatarsal with a non-displaced fracture line (Figure 4).

**Figure 5:**
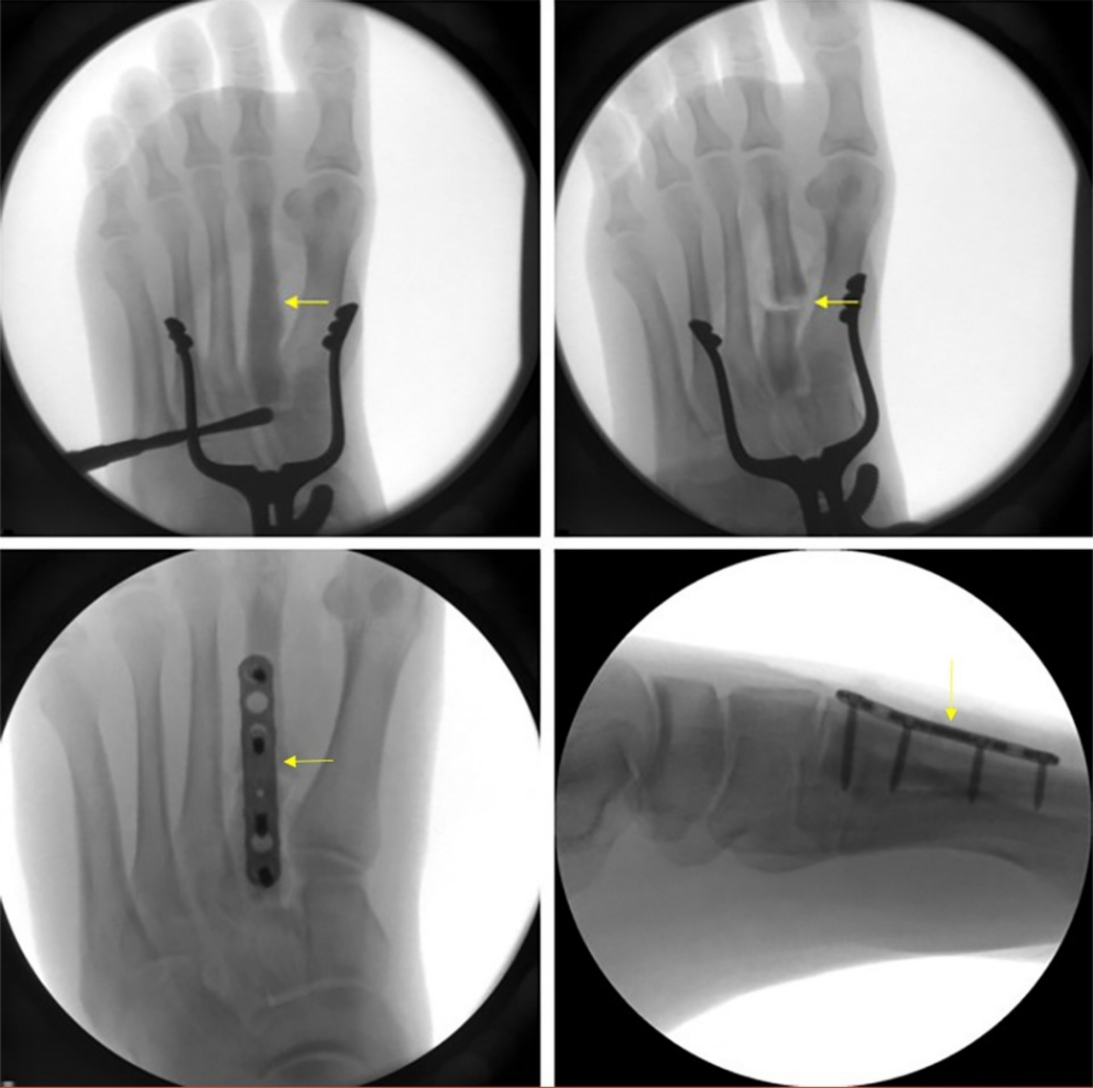
Intraoperative X-rays. Top row, left to right: cement in second metatarsal prior to open reduction and internal fixation; osteotomized second metatarsal in preparation for curettage and fixation. Bottom row, left to right: coronal view of metatarsal fixed with plate and screws; sagittal view of metatarsal fixed with plate and screws.

**Figure 6:**
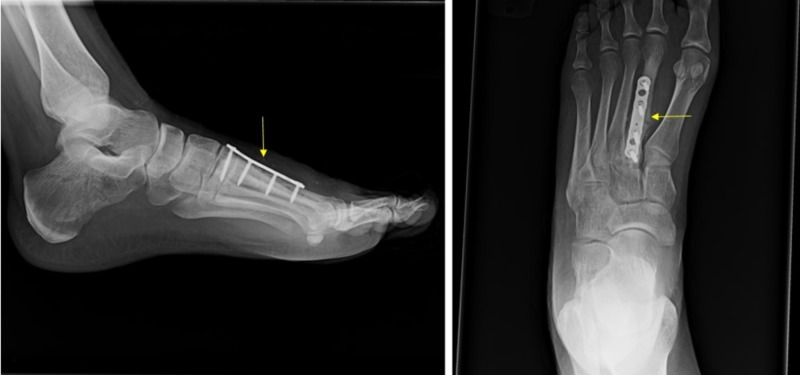
X-rays two weeks post-revision open reduction and internal fixation with plate and screws.

All treatment options were discussed with the patient and she agreed with undergoing operative fixation. In the operating room, cultures and a bone biopsy of the left second metatarsal were taken. After performing an osteotomy, curettage was performed to remove the injected cement. Open reduction and internal fixation was performed utilizing a plate and calcaneal bone graft (Figure 5). The patient was discharged home on the same day with adequate pain control and a bone stimulator. X-rays taken at two weeks post-revision surgery are shown in Figure 6.

**Figure 7:**
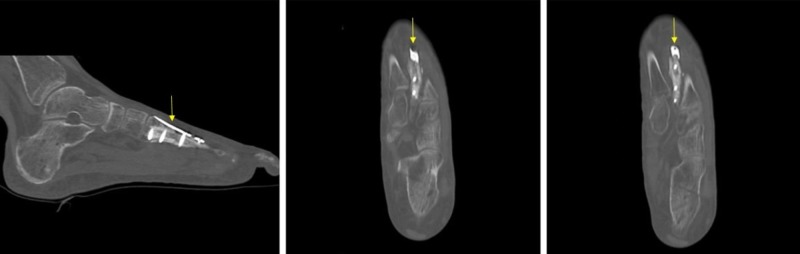
Post-operative computed tomography (CT) scan after revision open reduction and internal fixation with plate and screws.

At one-month follow-up, her incision was healing well without signs of infection and she had no complaints of pain. At her most recent appointment—three months post-revision surgery—she again reported no pain and good functional recovery with physical therapy. CT scan at three months post-revision surgery showed appropriate alignment of the healing second metatarsal with intact hardware (Figure 7).

## Discussion

Stress fractures of the foot most commonly affect the second and third metatarsals [6]. The second metatarsal accounts for as many as 52% of all metatarsal fractures. The goal of treatment is fracture stabilization to relieve pain and facilitate union [7]. Initial treatment of choice for most stress fractures of the second metatarsal is nonoperative [6, 8-10], as they are considered to be low-risk and typically heal well with conservative treatment. Management includes activity restriction, stiff-soled shoes [9], rest, ice, compression, elevation, and in some cases immobilization.

Operative treatment with open reduction and internal fixation is generally reserved for delayed union or nonunion [8-10], those who have dorsiflexion of the fracture [8], or fifth metatarsal fractures [11]. Surgery may also be considered first line in patients who desire to return to activity more quickly, such as athletes [10, 11]. For cases in which earlier weight-bearing and activity are desired, augmentation with bone graft can be useful in facilitating healing and preventing nonunion [10]. Intramedullary screw fixation in fifth metatarsal fractures has been shown to reduce time to fracture union, decrease complication rates, and reduce time needed to return to normal activity [11]. There is paucity of literature regarding the operative management of stress fractures of the second metatarsal. Muscolo et al. report successful fixation of a proximal second metatarsal fracture with a compression plate and four cortical screws [12].

Injectable cements have become increasingly popular as a suitable bone graft substitute to facilitate bone regeneration. Calcium phosphate cements and calcium sulphate cements are the two most common types [13]. In this case, the patient’s second metatarsal was injected with a composite cement composed of hydroxyapatite (a type of calcium phosphate cement) and calcium sulfate. Larsson and Hannink reviewed the chemical and biomechanical properties of current bone substitute cements, which act through two phases. In one phase the cement is paste-like and viscous, which allows for easy injection into and filling of bone defects. In the second phase, the liquid hardens into microporous cement. The micropores serve as routes for bone remodeling. Over time, the cement is resorbed by osteoclasts as bone undergoes remodeling and healing [13].

Clinically, injectable cements have proven to be safe and effective treatment modalities for various pathologic conditions [7, 14, 15]. A meta-analysis by Bajammal et al. demonstrated that, among patients with distal radial fractures, femoral neck fractures, intertrochanteric fractures, tibial plateau fractures, and calcaneal fractures, use of calcium phosphate cement resulted in less pain and a reduced risk of loss of reduction when compared to those who received bone graft [16]. Of note, these studies utilized the cement as augmentation to fixation, rather than as stand-alone treatment as in this case. Fewer studies have evaluated the clinical uses and outcomes of calcium sulphate cement. Iundusi et al. demonstrated the efficacy of a calcium sulphate hydroxyapatite cement in the augmentation of tibial plateau fractures as it fills residual gaps following hardware fixation [17]. Rauschmann et al. reported that an injectable calcium sulphate and hydroxyapatite composite cement, similar to that used in this case, was effective for pain relief and improvement in quality of life for a cohort of patients with osteoporotic vertebral fractures [7]. Studies have also reported successful application of calcium sulphate cement in osteoplasty, in which bone lesions or voids secondary to trauma, infection, or neoplasm are filled [18, 19]. However, Anselmetti suggests the use of polymethyl methacrylate for malignant lesions and calcium phosphate cement for benign lesions [20]. Additionally, this patient did not have any true “void” or lesion to fill. Although calcium sulfate cement has a multitude of documented uses, to our knowledge there are no published cases of application for treatment of metatarsal stress fractures.

## Conclusions

This unusual case suggests that injectable composite calcium sulphate-hydroxyapatite cement alone is not an effective method for treatment of second metatarsal stress fractures. However, due to its successful application for a broad spectrum of other pathologies, it may be beneficial to further investigate the possibility of utilizing biologic cement for augmentation of traditional metatarsal fracture fixation.
